# ATM: Main Features, Signaling Pathways, and Its Diverse Roles in DNA Damage Response, Tumor Suppression, and Cancer Development

**DOI:** 10.3390/genes12060845

**Published:** 2021-05-30

**Authors:** Liem Minh Phan, Abdol-Hossein Rezaeian

**Affiliations:** 1 Department of Molecular & Cellular Oncology, The University of Texas MD Anderson Cancer Center, Houston, TX 77030, USA; 2 Department of Drug Discovery & Biomedical Sciences, College of Pharmacy, The University of South Carolina, Columbia, SC 29208, USA

**Keywords:** ATM, cancer, DNA damage repair, DNA damage response, oxidative sensing, autophagy, hypoxia, pexophagy, mitophagy, cellular homeostasis

## Abstract

ATM is among of the most critical initiators and coordinators of the DNA-damage response. ATM canonical and non-canonical signaling pathways involve hundreds of downstream targets that control many important cellular processes such as DNA damage repair, apoptosis, cell cycle arrest, metabolism, proliferation, oxidative sensing, among others. Of note, ATM is often considered a major tumor suppressor because of its ability to induce apoptosis and cell cycle arrest. However, in some advanced stage tumor cells, ATM signaling is increased and confers remarkable advantages for cancer cell survival, resistance to radiation and chemotherapy, biosynthesis, proliferation, and metastasis. This review focuses on addressing major characteristics, signaling pathways and especially the diverse roles of ATM in cellular homeostasis and cancer development.

## 1. Introduction

During the past three decades, the study of ATM (ataxia telangiectasia mutated) has played a central role in advancing our understanding of the mammalian DNA damage response, cancer initiation and progression as well as redox signaling pathways. Numerous recent publications additionally reported the varied roles and influences of ATM in multiple cellular processes, for instance, growth, metabolism, energy production, oxidative homeostasis, chromatin remodeling, and genomic integrity, which are all important processes in tumor development and progression. Although ATM should be considered as a tumor suppressor because its mutations often lead to cancer, there are mounting evidence showing that ATM’s functions and signaling pathways may also contribute to cancer cells’ resistance to radiation, chemotherapy, and even assist tumor progression in some scenarios. In this review, we will cover the major features, roles, and signaling pathways of ATM, especially its diverse roles in tumor suppression and cancer progression.

## 2. The Structure and Domain Mapping of ATM

The ATM gene consists of 66 exons and is located on chromosome 11 (11q22-23) [1]. In its active monomeric form, ATM has a large size of 370 kDa. ATM is an important member of the PI3K-related protein kinase family (PIKK) [2]. Other members of this family include DNA-dependent protein kinase catalytic subunit (DNA-PKcs), ataxia-telangiectasia and Rad3-related (ATR), mammalian target of rapamycin (mTOR), SMG-1, TRRAP, among others [3,4,5]. Similar to these members, ATM possesses a kinase domain whose structure shares significant homology to that of the phosphatidyl inositol 3 kinase (PI3K). Thanks to this domain, ATM is an active serine/threonine kinase. Besides, ATM also has a FAT (FRAP (FK506-binding protein 12-rapamycin-associated protein (mTOR)), ATM, TRAPP (Transformation/transcription domain-associated protein)) domain and a FATC domain that is located on its C-terminal [6]. In 2006, Jiang et al. additionally showed that the FATC domain was essential for ATM to interact with its partners for activation and control of its kinase activity [7].

**Figure 1:**
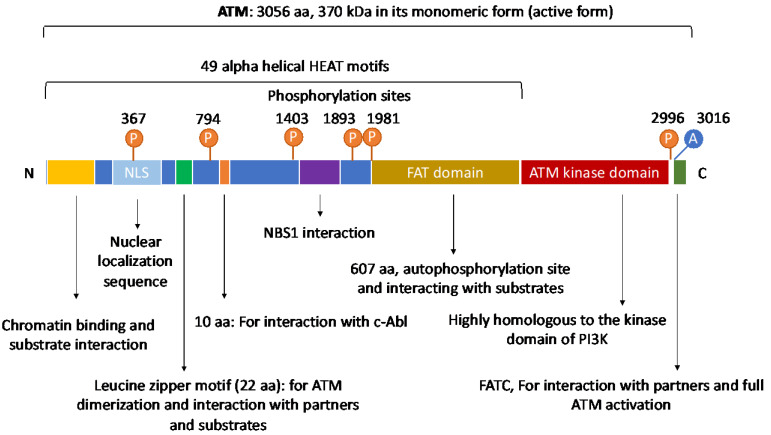
Human ATM’s domain map. *ATM* is a large gene with 66 exons and consists of multiple domains that allow its protein to directly interact with numerous regulators, partners, and downstream targets. In its N terminus, ATM contains a Nuclear Localization Signal that enables its nuclear translocation. ATM also uses its N-terminus for interacting with chromatin, substrates and partners, for instance, p53, LKB1, NBS1, among others. The putative leucine zipper motif on ATM N-terminus is documented to be essential for its dimerization and interaction with other partners or substrates. On its C-terminus, ATM has a FAT (FRAP, ATM and TRRAP proteins) domain which contains autophosphorylation sites and is critical for substrate binding. As a kinase, ATM has a serine/threonine kinase domain that is highly homologous to that of the PI3K. At the end of its C-terminus is the FATC domain that is essential for ATM full activation and interactions with partners. Of note, ATM contains multiple important autophosphorylation sites that can substantially affect its functions. For instance, Ser367, Ser1893, Ser1981, and Ser2996 are auto-phosphorylated after irradiation. Thr1885 is not induced by irradiation. In human cells, autophosphorylation on three residues Ser367, Ser1893, Ser1981, and acetylation on Lys3016 have been shown to be important for ATM activation. Aurora B phosphorylates ATM at Ser1403 during mitosis. Importantly, ATM also has 49 α helical HEAT motifs whose name is derived from huntingtin, elongation factor 3, the A subunit of protein phosphatase 2A, and target of rapamycin 1 (TOR1). These motifs serve as scaffolds and are critical for ATM interactions with proteins and DNA.

The N-terminus of ATM contains multiple α helical HEAT repeat motifs and a region that appear to be critical for ATM interactions with other proteins and DNA by serving as scaffolds. It has been shown that HEAT motifs are necessary for ATM to interact with and recruit a number of proteins to the DNA lesion sites for DNA damage repair [8,9,10,11]. In fact, ATMIN (ATM interacting protein), NBS1 (a component of the MRN complex), and NKX3.1 (a prostate tumor suppressor) have been found to bind to ATM’s HEAT motifs [12,13,14,15]. In 2004, Lavin et al., also reported the presence of other motifs, a proline-rich area for c-Abl binding, and a leucine zipper on ATM [16,17] (Figure 1).

## 3. ATM’s role in DNA Damage Response and Ataxia Telangiectasia

**Figure 2:**
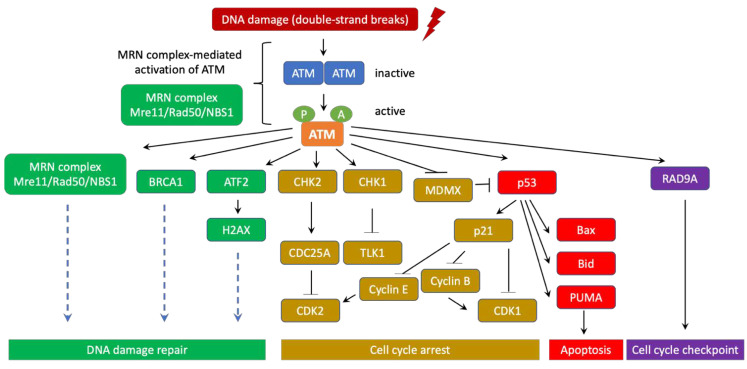
ATM canonical signaling pathway in DNA damage repair, cell cycle arrest and apoptosis. In normal situations, ATM are often inert and form homodimers or polymers. However, when DNA damage occurs, ATM is quickly activated and disassociates into highly active monomers. ATM initiates the DNA-damage response through co-signaling with the MRN (Mre11/Rad50/NBS1) complex at the DNA lesion sites. In fact, direct interaction with the MRN complex induces ATM to phosphorylate a number of downstream targets that are essential for DNA damage repair, cell cycle checkpoint, cell cycle arrest, and apoptosis. During this process, ATM undergoes a series of autophosphorylation on Ser367, Ser1893, Ser1981, and Ser2996 as well as acetylation on Lys3016. Activated ATM is recruited to the DNA damage sites for starting the DNA-damage response. ATM activates BRCA1 and ATF2 to promote cascades of DNA damage repair signaling pathways that involve hundreds of sensors, transducers, and effectors. In addition, ATM also turns on and stabilizes p53 via direct phosphorylation. ATM additionally phosphorylates MDMX to induce its degradation, thereby further stabilizing p53. To its turn, p53 translocates into the nucleus to transactivate a series of its downstream tumor suppressor target genes. For instance, p53 enhances the expression of p21, a potent cyclin-dependent kinase inhibitor. p21 associates with cyclin E, CDK2, CDK4/6 and induces G1/S and G2/M cell cycle arrest, which is critical to prevent unrepaired DNA mutations from passing into daughter cells. p53 also directly transactivates Bid, Bax, PUMA to induce apoptosis when DNA damage is too severe for effective repair. This programmed cell death is a major mechanism of tumor suppression. Moreover, ATM activates RAD9A to further promote cell cycle checkpoints. CHK1 and CHK2 are additionally induced by ATM through phosphorylation. CHK1 subsequently inhibits TLK1 while CHK2 turns on the cell cycle inhibitor CDC25A to block CDK2. As a result, cell cycle progression is temporarily halted to enable DNA damage repair. Thus, thanks to its central role in coordinating DNA damage repair, cell cycle arrest, cell cycle checkpoint, and apoptosis, ATM is frequently considered a major tumor suppressor whose mutations often lead to significant increase in risk of cancer.

ATM is an important initiator of the DNA-damage response in mammalian cells via the Mre11/Rad50/Nbs1 (MRN) complex at the DNA lesion sites [18,19,20,21]. ATM activation occurs after its direct binding to the MRN complex, leading to its conformational change from a resting homodimer or polymer state to an active monomer form [22]. Active ATM then uses its kinase activity to phosphorylate a number of downstream targets that are essential for DNA damage repair, apoptosis, cell cycle arrest, and cell-cycle checkpoints [21,23,24,25]. Therefore, ATM is often considered a major tumor suppressor (Figure 2).

Mutations of the *ATM* gene leads to deficiencies in the DNA-damage response, which results in the development of ataxia telangiectasia, a rare hereditary autosomal recessive disorder with a 1 in 40,000 to 300,000 frequency in Caucasians [26]. This condition is characterized by radiosensitivity, cancer predisposition, immunodeficiency (frequent infections), telangiectasias of the conjunctivae, chreoathetosis, and progressive neurodegeneration (cerebellar ataxia, oculomotor apraxia) [27,28,29,30,31,32]. In 1988, Gatti et al. used genetic linkage and found the location of the gene responsible for ataxia-telangiectasia on chromosome 11q [30]. In 1995, Savitsky et al. applied positional cloning and identified the gene *ATM* (ataxia-telangiectasia mutated) [1]. Of note, most of ataxia-telangiectasia patients carry hereditary heterozygote or homozygote *ATM* mutations from each parent. More than 300 *ATM* mutations have been identified and most of them involve insertions, deletions and base substitutions that result in abnormal mRNA splicing or premature termination codons. *ATM* mutations prevalence was estimated to vary from 0.5% to 1% in Caucasian populations, according to Swift et al., 1986 and Renwick et al., 2006 [26,33].

## 4. ATM Signaling Pathways

ATM signaling pathways could be classified into the canonical pathway and a diverse array of non-canonical pathways. In the canonical pathway, ATM collaborates with the MRN complex (Mre11/Rad50/NBS1) to activate the cellular DNA-damage response. ATM also uses a number of other non-canonical pathways to respond to other types of cellular stresses [34].

Similar to other PI3K family members, ATM kinase activity is strictly autoinhibited during its resting state (dimers or polymers) and only active when ATM binds to its partners. In response to DNA double-strand breaks (DSBs), ATM canonical pathway is induced, which involves the disassociation of ATM from dimers to monomers, activation and recruitment of ATM monomers to the DNA damage sites. It has been well documented that the MRN complex is required for ATM activation and recruitment to DSB locations. Indeed, the NBS1 subunit of the MRN complex directly binds to ATM and NBS1 ubiquitination promotes ATM recruitment to DSBs [18,20,22,35]. Subsequently, ATM phosphorylates a number of downstream targets as CHK1, CHK2, p53, BRCA1, ATF2, among others to stimulate DNA damage repair machinery [21,24].

In human, autophosphorylation at Ser1981 is often considered as a marker for ATM activation, which often occurs quickly after ionizing radiation [22]. In the absence of the MRN complex, ATM Ser1981 is not phosphorylated but ATM is still able to phosphorylate the downstream histone protein H2AX after radiation [12]. It is postulated that while phosphorylation at Ser1981 is not required for ATM function, this phosphorylation is critical to retain ATM at the DSBs [23,36]. Subsequent to H2AX phosphorylation, DNA repair complexes containing effectors, polymerases, among others are recruited to the DSB site. For instance, after being phosphorylated by ATM, MDC1 recruits RNF8, RNF168 [37,38,39], which in turn monoubiquitinates histone H2AX and H2A on Lys13 and Lys15 to prepare them for additional poly-ubiquitination reactions. These posttranslational protein modifications are necessary to recruit additional effector repair proteins [40]. It has been shown that continuous ATM activation is critical to maintain the damage foci for sustaining effective DNA damage repair [41]. It is important to point out that ATM-mediated signaling for DNA damage repair is tightly regulated by several feedback mechanisms to ensure proper and effective repair efficiency. For instance, it has been shown that due to the feedback of p53, ATM activation occurs in a pulse manner [42].

Chromatin decompaction and relaxation at DSB sites are essential for effective repair and ATM activation. In fact, ATM phosphorylates RNF20/RNF40 to facilitate histone H2B ubiquitination. ATM-mediated phosphorylation of KAP1 also decreases KAP1′s binding to CHD3, a chromodomain protein. These two events result in chromatin decompaction [43,44,45]. Of note, histone deacetylase inhibitors have been shown to activate ATM [22]. The histone acetyltransferase complex TIP60 is necessary for ATM activation after irradiation [46]. Interestingly, by increasing H3K14 acetylation and loosening chromatin structure, HMGN1 significantly elevates the amount of chromatin-bound ATM at DSB sites [47]. In contrast, to silence transcription at and near DNA damage sites, ATM promotes Histone H2A K119 mono-ubiquitination by BMI1 [48,49,50].

**Figure 3:**
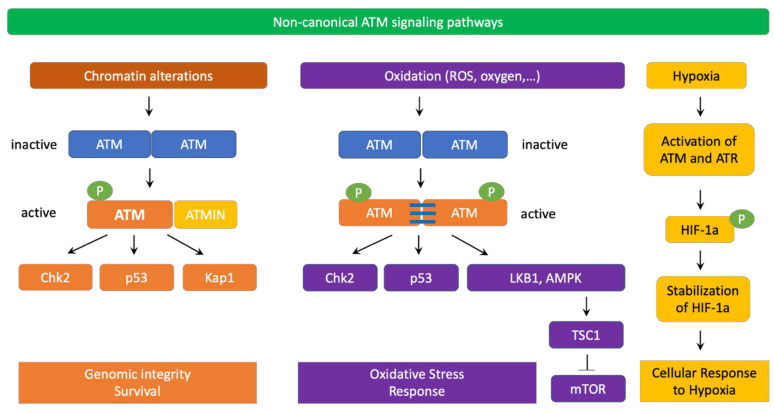
ATM non-canonical signaling pathways. Chromatin alterations induced by chloroquine or hypotonic stress could trigger ATM activation without requiring the MRN complex. The active monomer of ATM interacts with ATMIN (ATM interacting protein) to transduce downstream signaling to CHK2, p53, KAP1, and other substrates to promote genomic integrity and survival. ATM also serves as an important redox sensor. After being activated by ROS or oxidation, ATM homodimers establish disulfide bonds to maintain their dimer conformations. This activation process also does not require the MRN complex. Similar to active ATM monomers, induced ATM dimers are phosphorylated at Ser1981 but this posttranslational modification is not required for phosphorylation of downstream targets such as CHK2 at Thr68 or p53 at Ser18. Oxidation-activated ATM dimers then phosphorylate LKB1 and AMPK to turn on TSC2 and block mTOR signaling, thereby decreasing ROS levels. ATM can also be induced by severe hypoxia in a ROS-independent manner. In severe hypoxia, the function of ribonucleotide reductase is inhibited, causing the depletion of deoxynucleoside triphosphates (dNTPs) as well as replication stress. As a result, ATM and ATR are activated due to severe hypoxia. ATM and ATR then phosphorylate and stabilize HIF1α to enable cell survival under hypoxic conditions.

In non-canonical signaling pathways, ATM can also be activated by chromatin changes caused by chloroquine, hypotonic cellular stress, among others [22,51] (Figure 3). Interestingly, the MRN complex, especially NBS1, is not required for ATM non-canonical activation and signaling. Instead, ATMIN (ATM interacting protein) is required for ATM activation in these scenarios. Interestingly, ATMIN binds to ATM using interacting domains that are similar to those of NBS1 [52]. ATMIN is also partially required for ATM-mediated phosphorylation of downstream targets in non-canonical ATM pathways [53]. Furthermore, ATM also cross-talks with ATR for activation in response to UV radiation [54]. Importantly, in non-replicating cells, ATM can be activated by R-loops at transcription-blocking lesions. In fact, in 2015, Tresini et al., discovered that RNA polymerase pausing at the DNA damaged sites led to spliceosome displacement and the formation of R-loops, which subsequently activated ATM. R-loop-mediated activation of ATM prevented further spliceosome organization and increased genome-wide ultraviolet-irradiation-induced alternative splicing [55].

ATM is also involved in sensing oxidative stress. In this case, ATM dimers are oxidized and establish disulphide bonds between ATM monomers to create active ATM dimers and phosphorylate downstream targets. Exposure to oxygen at atmospheric levels or reactive oxygen species (ROS) activate ATM without requiring the MRN complex [56]. Active ATM dimers also carry phosphorylated Ser1981 but this phosphorylation is dispensable for ATM dimers’ function. Interestingly, after being induced by ROS, ATM dimers activate TSC2 by phosphorylating LKB1 and AMPK, thereby blocking mTOR signaling and reducing ROS production [57]. Importantly, ATM is activated by hypoxia and phosphorylates the transcription factor HIF1α, which leads to HIF1α stabilization and REDD1-mediated inhibition of mTOR [58]. Indeed, severe hypoxia impedes the activity of ribonucleotide reductase, leading to the depletion of dNTPs and subsequent replication stress. As a result, activation of ATM and ATR occurs. ATM and ATR then directly phosphorylate and stabilize HIF1α to promote cell survival under severe hypoxic conditions [59,60,61,62]. In 2017, Rezaeian et al. also showed that ATM indirectly induced HIF1α stability through H2AX phosphorylation in the cross talk with TRAF6-mediated H2AX ubiquitination [63]. As hypoxia and increased ROS stress frequently occur in solid tumors, ATM may promote cancer cell survival in some scenarios by stabilizing HIF1α and reducing ROS levels.

Recent studies additionally revealed a connection between ATM and autophagy [64,65,66] in response to nutrient deprivation, ROS, and DNA damage. Indeed, after being activated by oxidative stress or genotoxic agents, ATM suppresses mTORC1 while inducing autophagy [57,67,68]. ATM also increases the expression of *ATG4C* at the mRNA and protein levels, promoting autophagy in breast cancer stem cells [69]. Furthermore, ATM participates in regulating pexophagy in response to ROS [70,71]. Pexophagy is a catabolic process to selectively degrade peroxisomes by autophagy where autophagosomes engulf peroxisomes and fuse with lysosomes for subsequent degradation of peroxisomes. Pexophagy is critical to maintain cellular homeostasis by removing damaged or unnecessary peroxisomes. In the case of pexophagy, ATM is localized to peroxisomes through the PEX5 import receptor, which recognizes an SRL sequence on ATM’s C-terminus. As a result of ROS-mediated activation, ATM phosphorylates TSC2 to inhibit mTORC1, and ULK1 to promote autophagy. Furthermore, ATM directly phosphorylates PEX5 at Ser141. This phosphorylation subsequently leads to PEX5 ubiquitination at Lys209 and association with SQSTM1/p62 (an autophagy receptor) to trigger pexophagy [70,71]. In addition to genotoxic and oxidative stresses, ATM can be also activated by starvation and reactive nitrogen species [72]. Cytoplasmic ATM presence have been found in mitochondria peroxisomes, and endosomes, where the kinase exerts its regulatory functions in sensing oxidative stress and participate in the regulation of cellular metabolism and autophagy processes [73]. Some early evidence suggested that ATM helps maintain cellular homeostasis in response to DNA damage and ROS via the autophagy-senescence signaling axis [74]. Moreover, ATM has been documented to regulate mitophagy, a process to selectively remove mitochondria at the autophagolysosomes for turning over the dysfunctional or damaged mitochondria for adapting to physiological stress conditions, such as nutrient deprivation, hypoxia, DNA damage, among others. Recent evidences showed that ATM modulated Beclin-1 to control mitophagy [75,76] and mediated spermidine-induced mitophagy via regulating PINK expression and Parkin localization to mitochondria [77]. Together, these findings indicate an important role of ATM in sensing oxidative stress as well as regulating autophagy, mitophagy, and pexophagy to maintain cellular homeostasis.

## 5. ATM Signaling in Cancer

ATM’s major tumor suppressing mechanisms are inducing apoptosis and cell cycle arrest via activating p53, SIRT1, CHK1, CHK2, DBC1, RAIDD and other downstream targets [34]. Therefore, cancer cells can use different mechanisms to downregulate ATM. For instance, in breast cancer cells, ATM expression can be reduced due to miRNA-18a [78,79]. ATM activity could also be suppressed by the phosphatase WIP1, which directly dephosphorylates ATM and p53 [80].

Interestingly, in some tumor cells, ATM signaling and function are upregulated. Perhaps, those cancer cells have developed mechanisms to escape ATM-induced cell cycle arrest and apoptosis. Indeed, prostate cancer cells promote ATM expression via recruitment of the androgen receptor to the *ATM* gene enhancer region [81]. Pancreatic cancer cells increase *ATM* expression by overexpressing the transcription factor CUX1 [82,83]. Melanoma cells increase ATM signaling by increasing MAGE-C2 levels, which associates with KAP1 and promotes its Ser824 phosphorylation [84].

**Figure 4:**
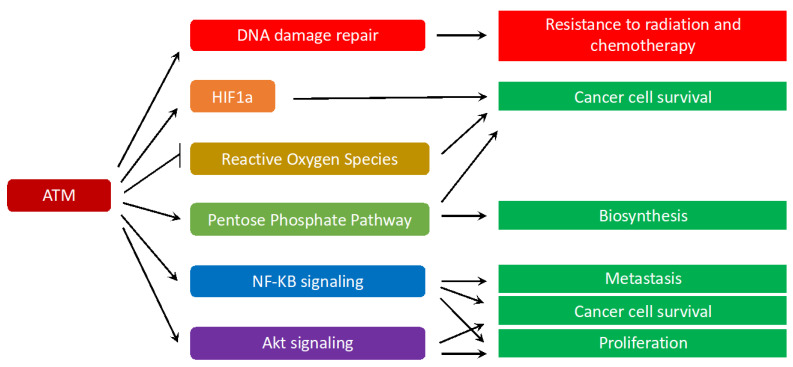
The diverse roles of ATM in cancer cells. In some cancer cells that have developed mechanisms to evade apoptosis and cell cycle arrest, increase in ATM signaling may confers significant advantages for cancer cell survival, biosynthesis, proliferation, metastasis, as well as resistance to radiation and chemotherapy.

Promoting ATM signaling and expression may facilitate cancer cells’ resistance to chemotherapy and radiation, metastasis, and tumor cell survival [79] (Figure 4). As radiation therapy and many chemotherapy agents attack cancer cells by causing DSBs, elevated ATM’s function and signaling provide tumors with significant advantages to adapt and survive these treatments. In fact, ATM-mediated activation of Akt can induce cancer cell survival in certain scenarios [82]. NF-KB activation by ATM additionally increases cancer cell survival, blocks apoptosis, and facilitates Epithelial-Mesenchymal Transition, as well as cancer cell migration and metastasis [85]. ATM also triggers the pentose phosphate pathway to reduce ROS levels. This metabolic pathway also provides cancer cells with essential intermediates for biosynthesis and proliferation [86,87,88]. Of note, increased ATM function has been found to be associated with elevated metastasis, invasion, and Epithelial-Mesenchymal Transition of breast cancer cells that overexpress HOXB9 or under-express PRSS11 [89,90,91]. Moreover, ATM is activated by hypoxia, and stabilizes the transcription factor HIF1α by direct phosphorylation or via the TRAF6/H2AX/HIF1α signaling axis, which could significantly increase cancer survival, invasion, and metastasis [58,63,79].

## 6. Conclusions

Numerous studies during the past three decades show that ATM is a central coordinator of the DNA-damage response and plays a vital role in maintaining genomic integrity and suppressing cancer at early stages. Indeed, ATM suppresses tumors by inducing apoptosis and cell cycle arrest. However, in some cancer cells that have already escaped those tumor-suppressing mechanisms, ATM signaling may be beneficial for cancer survival, resistance to chemotherapy and radiation, promoting Epithelial-Mesenchymal Transition, proliferation, invasion, and metastasis. Therefore, ATM-based anti-cancer therapy should be carefully selected and tailored based on the characteristics of the tumors to achieve significant and long-lasting therapeutic effects. Interestingly, beside its functions in DNA damage response and maintaining genomic integrity, ATM is also involved in oxidative stress sensing, regulating autophagy, mitophagy, pexophagy, and maintaining cellular homeostasis in response to ROS, starvation, and hypoxia. Together, these findings suggest a multi-faceted role of ATM in various cellular processes, which significantly expands its functions beyond guarding genome stability.

## Data Availability

Not applicable.
